# Optogenetic Activation of β-Endorphin Terminals in the Medial Preoptic Nucleus Regulates Female Sexual Receptivity

**DOI:** 10.1523/ENEURO.0315-19.2019

**Published:** 2020-01-22

**Authors:** Caroline Johnson, Weizhe Hong, Paul Micevych

**Affiliations:** 1 Department of Neurobiology David Geffen School of Medicine at University of California, Los Angeles, Los Angeles, CA 90095; 2 Laboratory of Neuroendocrinology of the Brain Research Institute, University of California, Los Angeles, Los Angeles, CA 90095; 3 Department of Biological Chemistry, David Geffen School of Medicine, University of California, Los Angeles, Los Angeles, CA 90095

**Keywords:** β-endorphin, estradiol, lordosis, μ-opioid receptor, POMC

## Abstract

Estrogen and progesterone (P4) act in neural circuits to elicit lordosis, the stereotypical female sexual receptivity behavior. Estradiol acts through membrane receptors to rapidly activate a limbic-hypothalamic circuit consisting of the arcuate (ARH), medial preoptic (MPN), and ventromedial (VMH) nuclei of the hypothalamus. This initial activation results in a transient but necessary inhibition of lordosis, which appears to be a result of the release of β-endorphin (β-End) from proopiomelanocortin (POMC) terminals onto cells containing the µ-opioid receptor (MOR) in the MPN. To functionally examine the role of the MOR in the hypothalamic lordosis circuit, we transfected a channelrhodopsin (ChR2) adeno-associated virus into POMC cell bodies in the ARH and photostimulated POMC/β-End axon terminals in the MPN in sexually receptive female Pomc-cre mice. Following estrogen and P4 priming, sexual receptivity was assessed by measuring the lordosis quotient (LQ). Following an initial trial for sexual receptivity, mice were photostimulated during behavioral testing, and brains were processed for MOR immunohistochemistry (IHC). Photostimulation decreased the LQ only in ChR2-expressing Pomc-cre mice. Furthermore, photostimulation of ChR2 in POMC/β-End axon terminals in the MPN resulted in the internalization of MOR, indicating activation of the receptor. Our results suggest that the activation of the MOR in the MPN is sufficient to attenuate lordosis behavior in a hormone-primed, sexually receptive female mouse. These data support a central role of MOR in female sexual behavior, and provide further insight into the hypothalamus control of sexual receptivity.

## Significance Statement

The µ-opioid receptor (MOR) has been shown to modify lordosis, the female sexual receptivity behavior. Estrogen and progesterone (P4) facilitate lordosis, though the initial effects of estradiol in the arcuate nucleus of the hypothalamus (ARH) inhibit the behavior through MOR activity in the medial preoptic nucleus (MPN). The present study uses optogenetics to directly stimulate proopiomelanocortin (POMC)/β-endorphin (β-End) axonal terminals in the MPN in sexually receptive female mice to examine the effects of MOR activity on lordosis. Photostimulation of these terminals is sufficient to activate and externalize the MOR, and to attenuate lordosis behavior in otherwise sexually receptive mice. Our results provide further evidence of an ARH to MPN projection that, by activating MOR, is inhibitory to lordosis behavior.

## Introduction

Innate motivated behaviors are crucial for the survival of the individual (e.g., eating, drinking) or the species (e.g., reproduction). Reproduction is dependent on the sexual receptivity of the female, which in many species is regulated by ovarian steroids at specific times. Lordosis, female sexual receptivity behavior, is a reflexive behavior in which the female arches the spine, elevates the hind quarters, and lifts the tail and head to allow the male to intromit (Beach, 1948; Pfaff et al., 1994). In cycling females the release of ovarian hormones tightly regulates receptivity through the priming of specific neural circuits along with appropriate sensory cues (Micevych and Sinchak, 2007), to ensure that lordosis occurs in response to mounting by a conspecific male (Micevych and Ulibarri, 1992; Micevych and Sinchak, 2007) at a time in the cycle that maximizes the potential for reproductive success. While the manifestation of such a behavior involves many sites along the neuraxis, a few critical circuits have emerged that are involved in the production of lordosis behavior, including mesolimbic and hypothalamic-limbic circuits (Micevych and Sinchak, 2007; Micevych and Meisel, 2017).

**Figure 1.**
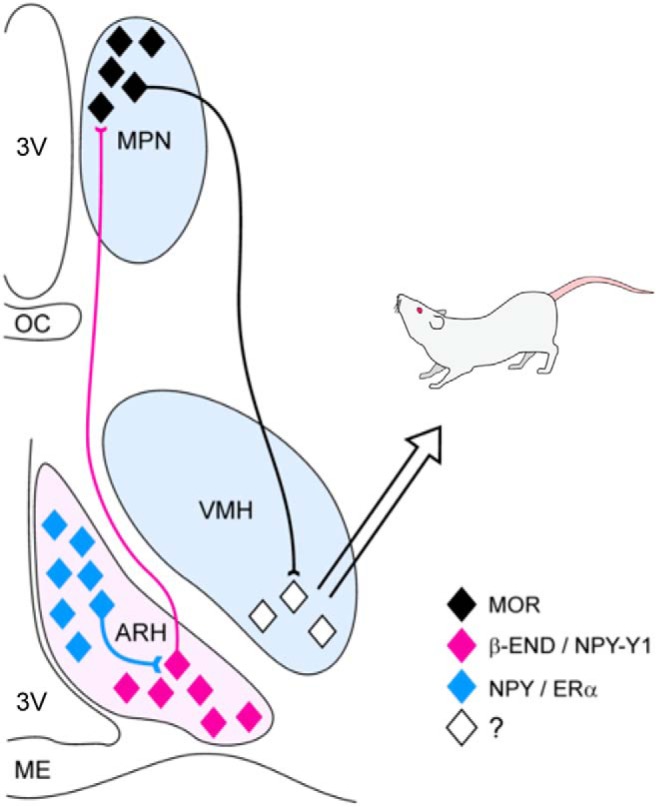
Hypothalamic lordosis circuit. Estradiol acts initially on NPY neurons in the ARH, which project to and activate POMC/β-End neurons. These neurons project to the MPN where the release of β-End activates and internalizes MORs. When these receptors are internalized, lordosis is attenuated. Neurons from the MPN project further to the VMH, where signals from other circuits are integrated before projecting to lower brain regions and ultimately to the spinal motor neurons responsible for the manifestation of lordosis behavior. 3V, 3rd ventricle; OC, optic chiasm; ME, median eminence. Figure adapted from Micevych and Christensen (2012), with permission.

The circuit consisting of the arcuate nucleus (ARH), medial preoptic nucleus (MPN), and ventromedial nucleus (VMH) of the hypothalamus has been shown to be vital for the expression of lordosis (Sinchak and Micevych, 2001; Micevych and Meisel, 2017; Fig. 1). A subset of neuropeptide Y (NPY) and proopiomelanocortin (POMC) neurons located in the ARH express estrogen receptor (ER)α (Sar et al., 1990; Simonian et al., 1999) and are involved in reproduction (Cheung and Hammer, 1995; Cheung et al., 1995). Indeed, estradiol initially acts in the ARH to activate both NPY and POMC neurons. Previous work has indicated that estradiol treatment activates NPY-Y1 receptors on POMC/β-endorphin (β-End) neurons within the ARH (Mills et al., 2004).

The POMC population within the ARH is highly heterogeneous, both in physiology and function (Cheung and Hammer, 1995; Ibrahim et al., 2003; Hentges et al., 2009; Campbell et al., 2017; Lam et al., 2017), and this heterogeneity makes parsing out the different roles of these neurons difficult. POMC neurons span the rostral-caudal extent of the ARH and project to various nuclei throughout the hypothalamus and surrounding regions (Sinchak and Micevych, 2003; Smyth, 2016). The rostral ARH POMC population that is involved in reproduction appears to be separate from that involved in food intake, distinguished by distinct morphologic properties (Cheung and Hammer, 1995), as well as physiologic properties (Ibrahim et al., 2003). These reproductively important POMC neurons project to the medial portion of the MPN, where there is a concentration of cells that express µ-opioid receptors (MORs; Simerly and Swanson, 1986; Cheung and Hammer, 1995; Eckersell et al., 1998; Mills et al., 2004 ).

MORs have been implicated in regulating sexual receptivity in rodents (Torii et al., 1999; Sinchak and Micevych, 2001; Acosta-Martinez and Etgen, 2002a; Mills et al., 2004; Micevych et al., 2017), mediated by the release of the endogenous opioid peptide β-End, which has a high affinity for MOR (Birdsall and Hulme, 1976). Activation of MORs can be monitored by examining their cellular location; MOR-immunoreactivity (MOR-ir) localized primarily on the cell surface indicates receptors that have not been activated, while activated MOR are rapidly internalized and appear in the cytoplasm. The initial actions of estradiol within this circuit result in a transient but necessary inhibition of lordosis, through the activation and internalization of MOR in the MPN (Eckersell et al., 1998; Sinchak and Micevych, 2001; for review, see Micevych et al., 2017).

The estradiol activation of MOR is dependent on ERα (Micevych et al., 2003). MOR activation is out of phase with sexual receptivity throughout the estrous cycle (Sinchak and Micevych, 2003); when receptors are engaged lordosis is inhibited, and reversal of this activation by progesterone (P4) allows lordosis behavior (Sinchak and Micevych, 2001; for review, see Micevych and Meisel, 2017). MPN neurons project to the VMH (Simerly and Swanson, 1988), the final integrative hub for lordosis behavior within the hypothalamus. From here, projections innervate lower brain regions and eventually the spinal motor neurons to produce the behavior (for review, see Micevych et al., 2015; Micevych and Meisel, 2017).

We tested whether optogenetic activation of β-End terminals only in the medial MPN would lead to the activation/internalization of MOR and inhibition of sexual receptivity. To this end, channelrhodopsin (ChR2)-expressing AAVs were inserted into Pomc-cre neurons in the ARH, followed by selective photostimulation of the β-End-releasing terminals in the MPN in hormone-primed, sexually receptive female mice, and lordosis behavior was examined.

## Materials and Methods

### Animals

Adult [postnatal day (P)60] female Pomc-cre mice [originally JAX #005965 (Balthasar et al., 2004) and #010714 (McHugh et al., 2007)] from our colony, and adult (P60) male C57/Bl6J from Charles River (Charles River Laboratories, Inc.) were used for all experiments. All female mice received ovariectomies and fiber optic cannulae (see below, Surgeries). Mice were randomly assigned to one of four groups: (1) ChR2-AAV + hormone replacement, (2) control AAV + hormone replacement, (3) ChR2-AAV + oil control, or (4) control AAV + oil control. An additional control group was cre-positive mice that received the ChR2-AAV and hormone replacement, but in which the fiber optic cannulae was incorrectly placed. See Table 1 for group descriptions and Table 2 for the number of mice of each strain per group. All animal procedures were performed in accordance with the regulations of the University of California-Los Angeles Chancellor’s Animal Research Committee.

**Table 1.** Experimental group parameters

Group	Virus	Hormone replacement	*n*
Group 1	ChR2	EB+P4	7
Group 2	Control	EB+P4	6
Group 3	ChR2	Oil	4
Group 4	Control	Oil	4
Group 5	ChR2	EB+P4	4

Presented are the groups of mice used in this study, including AAV, hormone treatment, and number of mice per group. All hormones were dissolved in safflower oil and delivered subcutaneously 2 h before lights off. Mice that did not receive hormone replacement instead received an equal volume of safflower oil only.

**Table 2.** Transgenic strains used in study

	*n*	
Group	JAX #005965	JAX #010714
Group 1	6	1
Group 2	4	2
Group 3	2	2
Group 4	2	2
Group 5	4	0

Two strains of Pomc-cre mice were used in this study; provided are the number of each strain of mouse used in each experimental group (described in Table 1).

### Surgeries

Adult (P60) female Pomc-cre mice were anesthetized under isoflurane and transfected with an AAV expressing ChR2 (AAV1.CAGGS. Flex.ChR2-tdTomato.WPRE.SV40; Addgene catalog #18917-AAV1) or the control virus (AAV1.CAG.Flex.tdTomato.WPRE.bGH; Addgene catalog #28306-AAV1). AAVs were delivered bilaterally into the ARH (from bregma; AP: –1.50, ML: ±0.25, DV: –5.50) with a 5-µl Hamilton syringe (Hamilton Company, #7634-01) equipped with a 32-G removable needle (Hamilton Company, #7803-04), via World Precision Instruments UltraMicroPump (World Precision Instruments, Inc., UMP3-3), at a rate of 30 nl/min for a total volume of 300 nl per side. AAVs were allowed five weeks to incubate to result in full fiber expression in the MPN before behavioral testing. Following injections, a custom-made ferrule fiber (200-µm core diameter, 240-µm outer diameter, Doric Lenses) was implanted into the MPN (from bregma; AP: 0, ML: ±0.35, DV: –4.75) and fixed on the skull with dental cement (Parkell, Metabond). Two weeks before behavioral testing, mice were bilaterally ovariectomized (ovx), to allow sufficient time for the loss of endogenous hormones.

### Hormone replacement

Mice received subcutaneous injections of 17β-estradiol benzoate (EB) and P4 dissolved in safflower oil, 2 h before lights out, over a 3-d period to mimic the estrous cycle. On day 1, mice received 20-µg EB, on day 2, 10-µg EB, and on day 3, 250-µg P4. Control mice received injections of equivalent volumes of safflower oil only.

### Behavioral and optogenetic testing

Lordosis behavior was tested 2 h after lights off on the third day of hormone injections. Before running experimental behavior tests, each female was exposed to a sexually experienced male once before behavior testing as training. Data from three subsequent sets of experimental behavioral tests were collected and averaged per test per animal. Approximately 15 min before behavior testing, sexually experienced adult male mice were placed in individual Plexiglas testing arenas to acclimate. Immediately before being placed in the arena with the male, the optogenetic patch cord (Doric Lenses) was attached to the implanted fiber optic cannula via a zirconia mating sleeve (Doric Lenses). The patch cord remained attached for the duration of the behavior test. A single round of behavioral testing consisted of two interactions with a male mouse, each interaction lasting for 10 mounts by the male. If the male ejaculated, the female was moved to a different arena with a different male to continue testing. For the first interaction of the behavior test, each female mouse was subject to a pre-test to determine sexual receptivity, measured by the lordosis quotient (LQ; the number of times a female displays lordosis/10 mounts by a male × 100). If the mouse displayed a LQ <50 it was considered unreceptive, and there was no further testing that day. Each test began when the female was placed in the cage and lasted until the male mounted the female 10 times. Following the pre-test, the female was removed from the arena and the laser was switched on for at least 5 min before the second test, to allow for release of the peptide and engagement of MORs before being placed back in the arena with the male. Photostimulation (ChR2, 473 nm, 20 Hz, 20-ms pulses, 1–3 mW/mm^−2^) was applied for the duration of second interaction with the male. Behavior tests were recorded using a Yi Action Camera (XiaoYi Technology Co., LTD) and scored by observers blinded to experimental condition. Following the final behavior test mice were perfused 60 min after removal from the arena. For each animal, the results of the three pre-tests were averaged to a single LQ score, as were the results of the three photostimulation tests.

### Perfusion, removal, and sectioning

Mice were transcardially perfused with cold 0.9% saline, followed by cold 4% paraformaldehyde (PFA) in Sorenson’s buffer (pH 7.4). Brains were removed and post-fixed in the same PFA solution for 24 h, then switched to 30% (w/v) sucrose in phosphate buffer overnight before being flash frozen in hexanes cooled on dry ice. Brains were sectioned 25 µm coronally using a Leica cryostat (Leica Biosystems, CM1950) and stored in a cryoprotectant solution at –20°C until used for immunohistochemistry (IHC).

### IHC

Sections containing the MPN were processed for MOR IHC, and sections containing the ARH were processed for cFOS IHC to verify POMC/β-End neuronal activation, via tyramide signal amplification (TSA) process. Sections were washed in tris-buffered saline (TBS; pH 7.4) on a rotating table for 30 min at room temperature (RT). Sections were transferred to a solution of 0.5% H_2_O_2_ in TBS for 10 min, then washed again in TBS for 30 min. Sections were then blo*c*ked in a solution of 2% normal goat serum (NGS; Equitech-Bio, #SG30-0500) and 0.03% Triton X-100 (Sigma-Aldrich, #X100-100ML) in TBS for 1 h at RT. Sections incubated for 1 h at RT in a 1% NGS/0.03% Triton X-100 solution, containing either rabbit anti-MOR or rabbit anti-cFOS (Table 3), before being placed on a rotating table at 4°C for 48 h. Sections were then washed in a solution of 0.03% Triton X-100 in TBS (TBST) for 30 min at RT, then transferred to a solution containing biotinylated goat anti-rabbit antibody, at 1:300 dilution, in TBST for 1 h. Sections were washed in TBST for 30 min as before, then incubated in a TBST solution containing 0.2% Solution A and 0.2% Solution B (VectaStain ABC HRP kit, #PK-4000, Vector Laboratories) for 1 h at RT. Sections were rinsed in TBS for 30 min, then transferred to a solution containing 0.6% biotinylated tyramide in a borate buffer. Sections were washed in TBS as before, then incubated in a TBST solution containing Streptavidin conjugated to Alexa Flour 488 (1:2000; Table 4). Sections were rinsed a final time in TBS, mounted onto SuperFrost slides (Fisher Scientific, #12-550-15). Once dry, slides were applied with mounting medium containing DAPI (DAPI Fluoromount-G, Southern Biotech, #0100-20) before being coverslipped and sealed with nail polish. Slides were stored in the dark at –20°C until imaging.

**Table 3.** Primary antibodies

Antibody	Dilution	Source
Rabbit anti-MOR	1:4000	Neuromics, #RA10104
Rabbit anti-cFOS	1:5000	Abcam, #ab190289
Rabbit anti-POMC	1:8000	Phoenix Pharmaceuticals Inc., #H-029-30

Provided is the species in which the antibody was raised, dilution of antibody in microliters, and commercial source of primary antibodies used.

**Table 4.** Secondary antibodies

Antibody	Dilution	Source
Biotinylated goat anti-rabbit IgG	1:300	Vector Laboratories, #BA-1000
Streptavidin Alex Fluor 488	1:2000	Molecular Probes, #S-11223
Alexa Fluor 488 donkey anti-rabbit IgG	1:2000	Jackson ImmunoResearch, #711-545-152

Provided is the type of secondary antibody including species in which it was raised, dilution of antibody in microliters, and commercial source.

To verify that neurons infected by the AAVs expressed POMC, IHC was performed using an antibody against POMC (1:8000; Table 3). Sections containing the ARH were washed 3 × 10 min in TBS and blocked in a solution of TBS/2% NDS/0.3% Triton X-100 for 1 h at RT. Sections were incubated in a solution containing rabbit anti-POMC at 4°C overnight. Sections were again washed at RT as before, then transferred to a solution containing the fluorescent secondary antibody donkey anti-rabbit Alexa Fluor 488 (1:2000; Table 4) and allowed to incubate for 2 h at RT. Sections were washed a final time before being mounted onto SuperFrost slides as above. See Tables 3, 4 for dilutions and commercial source of antibodies used.

### Imaging

Images were obtained with a Zeiss LSM880 (Zeiss Blue software, Zeiss) using the 405, 488, and 561 laser lines, with appropriate emission filters to prevent optical bleed through. Sections containing the MOR in the MPN were imaged with a 63× oil objective (NA 1.4), employing an optical zoom of 4.7 for a final magnification of ∼300. Three cells expressing MOR-ir in the dorsal part of the medial MPN were imaged at three Z-planes spanning the depth of the cell. Cells in the ARH expressing cFOS, and those labeled with the antibody against POMC, were imaged using a 10× (NA 0.45) or 20× (NA 0.75) objective to assess colocalization of cFOS-ir or POMC-ir and tdTomato.

### Data analysis

Images were analyzed using Imaris software (Imaris 9.2.1, Bitplane, Oxford Instruments Group). Previous studies (Dournaud et al., 1998; Eckersell et al., 1998; Sinchak and Micevych, 2001, 2003; Mills et al., 2004) have indicated a correlation between receptor internalization and the number of MOR-ir structures. To analyze MOR-ir and internalization, the Surfaces feature was used. A ROI containing only the cell of interest was analyzed, with a threshold for MOR-ir set at 1 SD above mean background fluorescent intensity, per image. The minimum size threshold to further exclude nonspecific for a labeled structure was set at 0.1 µm. Images were analyzed for the area of the image that contained MOR-ir, and divided by the total area of the image, to obtain the percentage of the image expressing MOR-ir. Each of the three Z-planes per cell was analyzed and averaged to find the total area covered per analyzed cell, and this process was repeated for the three total cells imaged per animal and averaged.

Colocalization of cFOS-ir and tdTomato, as well as POMC-ir and tdTomato, was analyzed using ImageJ (Schneider et al., 2012). The total number of cells in the ARH expressing tdTomato only, cFOS-ir or POMC-ir only, and both cFOS-ir or POMC-ir and tdTomato was counted. Additionally, the percentage of all cFOS-labeled cells expressing tdTomato, and the percentage of total tdTomato expressing cFOS or POMC, was analyzed. Images were optimized by adjusting the brightness uniformly across all pixels.

### Statistics

Statistics for behavioral and imaging data were analyzed using a one-way ANOVA or a two-way ANOVA, followed by Tukey’s multiple comparisons tests, as appropriate. All statistics were analyzed using GraphPad Prism version 7.03 for Windows (GraphPad Software). Significance for all analyses was set at *p* ≤ 0.05, and all values are expressed as mean ± SEM.

## Results

### AAV expression and fiber optic placement

**Figure 2.**
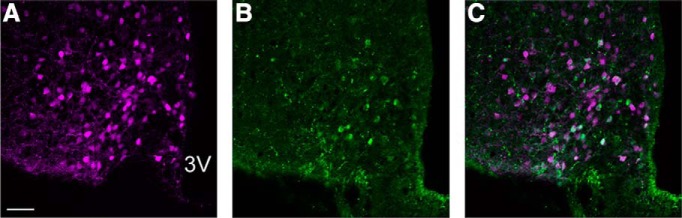
POMC expression in tdTomato-labeled cell bodies. IHC was run to confirm that tdTomato was expressed in POMC neurons in the ARH. ***A***, tdTomato in magenta. ***B***, Rabbit anti-POMC in green. ***C***, Merged image showing tdTomato and POMC-ir. 3V, 3rd ventricle. Scale bar = 50 µm.

POMC cell bodies were localized along the entire rostral-caudal extent of the ARH, in agreement with previous studies (Finley et al., 1981; Cheung and Hammer, 1995; Smyth, 2016). Along with projections to the MPN, fibers expressing tdTomato projected to various nuclei within the hypothalamus, amygdala, and bed nucleus of the stria terminals. Fibers rarely innervated the VMH. Transfection of POMC neurons was confirmed using IHC along with fluorescence microscopy, as both the ChR2-AAV and the control virus were conjugated to tdTomato. IHC was used to verify that neurons infected with tdTomato did in fact express POMC. In agreement with Padilla et al. (2012), we found that not all neurons that expressed tdTomato also expressed POMC-ir. In the present study, ∼30% of the tdTomato-positive neurons in the ARH also expressed POMC-ir, while nearly 70% of neurons expressing POMC-ir co-expressed tdTomato (Fig. 2).

**Figure 3.**
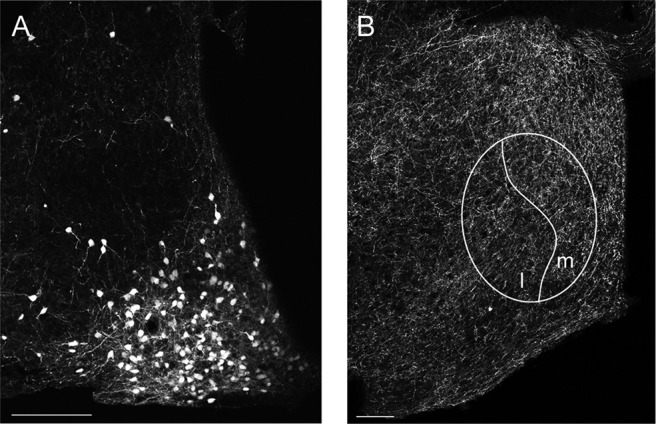
ChR2 expression in ARH and MPN. A representative image showing (***A***) soma and fiber expression of ChR2-tdTomato in the ARH, and (***B***) fiber expression in the MPN in the same animal. The MPN is shown at the level containing the medial (m) and lateral (l) subdivisions. 3V, 3rd ventricle. Scale bars = 100 µm.

Only mice in which both cell bodies in the ARH and axonal fibers in the MPN expressed tdTomato were included in the study. Cre-positive mice with no tdTomato expression were excluded from the experimental analyses. All mice showed expression of tdTomato in cell bodies in the ARH (Fig. 3*A*), and all mice expressed labeled fibers in the MPN (Fig. 3*B*).

In the MPN, fiber optic cannulae placement was histologically confirmed. A group of mice (*n* = 4) expressing ChR2 had misplaced fiber optic cannulae, and these animals were used as an additional control group (group 5; Table 1). All mice underwent a pre-test to determine sexual receptivity. Mice receiving hormone replacement with a pre-test LQ of <50 were excluded from the study. As expected, mice receiving oil only, without hormone replacement, were never receptive.

### Optogenetic stimulation of β-End in the MPN reduced sexual receptivity

**Figure 4.**
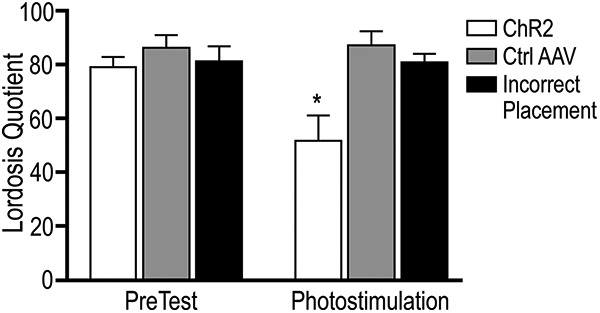
Photostimulation of ChR2 in the MPN significantly attenuates the LQ. A two-way ANOVA followed by Tukey’s multiple comparisons test indicated a significant interaction between virus type and behavior test on the LQ (*F*_(2,28)_ = 3.96, *p* = 0.03). Photostimulation of ChR2-expressing fibers in the MPN significantly reduced the LQ as compared to all other conditions (denoted by *). There was no difference in initial sexual receptivity between any of the three groups. In Pomc-cre mice expressing ChR2 in β-End terminals, photostimulation significantly attenuated lordosis (photostimulation, white bar) as compared to the pre-test without photostimulation (pre-test, white bar; simple main effect *p* = 0.01). Photostimulation of ChR2 (white bar) also significantly reduced the LQ as compared to both mice with the control virus (gray bar, simple main effect *p* = 0.001) and mice with incorrectly placed fiber optic cannulae (black bar, simple main effect *p* = 0.03). Photostimulation of ChR2 (photostimulation, white bar) furthermore significantly reduced the LQ as compared to the pre-test LQ of both mice receiving the control virus (pre-test, gray bar, simple main effect *p* = 0.002) and those with incorrectly placed fiber optic cannulae (pre-test, black bar, simple main effect *p* = 0.03). Mice that received the control AAV exhibited no difference (*p* > 0.99) in LQ between the pre-test and during photostimulation, nor did mice expressing ChR2 but with incorrect placement of the fiber optic cannula (*p* > 0.99). There was no difference in LQ between mice that received the control virus and those with ChR2 but incorrect placement of fiber optic cannulae in any condition. Values are expressed as mean ± SEM.

A two-way ANOVA followed by Tukey’s multiple comparisons post-test was conducted to compare the effect of virus and photostimulation on the LQ, and analysis showed a significant interaction (*F*_(2,28)_ = 3.96, *p* = 0.03; Fig. 4). There was a significant main effect of viral treatment (*F*_(2,28)_ = 7.73, *p* = 0.002). See Table 5 for pre-test group LQ means and SEM and Table 6 for group means and SEM following photostimulation.

**Table 5.** Mean LQ during pre-test

	Mean	SEM
Group 1	79.9	3.3
Group 2	86.8	4.5
Group 5	82.0	5.2

Presented is the mean and the SEM of the pre-test LQ score for each of the groups that received hormone replacement.

**Table 6.** Mean LQ following photostimulation

	Mean	SEM
Group 1	52.6	9.0
Group 2	88.0	4.8
Group 5	81.4	3.0

Presented is the mean and the SEM of the LQ score during photostimulation for each of the groups that received hormone replacement.

Tukey’s multiple comparisons test indicated that there was no difference in pre-test LQ between any of the three groups (group 1 vs group 2, simple main effect, *p* = 0.94; group 1 vs group 5, simple main effect, *p* = 0.99; group 2 vs group 5, simple main effect, *p* = 0.99; Fig. 4). Analysis of simple main effects revealed that overall, mice expressing ChR2 with properly placed fiber optic cannulae showed significant attenuation of the LQ as compared to all other conditions (Fig. 4). Compared with the pre-test LQ, photostimulation of fibers expressing ChR2 in the MPN significantly reduced the LQ (simple main effect, *p* = 0.01; Fig. 4) in those mice expressing ChR2 with correct cannula placement (group 1). Pomc-cre mice that received the control virus (group 2) showed no difference in LQ between the pre-test and during photostimulation (simple main effect, *p* > 0.99; Fig. 4), nor did those mice that expressed ChR2 but in which the fiber optic cannula was misplaced (group 5; simple main effect, *p* > 0.99; Fig. 4).

During photostimulation, mice expressing ChR2 (group 1) had significantly lower LQ scores than those expressing the control virus (group 2; simple main effect, *p* = 0.001; Fig. 4), a difference of ∼35%, similar to that seen between the pre-test and photostimulation conditions of ChR2 mice alone. Furthermore, only when the fiber optic cannulae were correctly positioned did photostimulation in mice expressing ChR2 attenuate the LQ (group 1 vs group 5, simple main effect, *p* = 0.03; Fig. 4). Photostimulation did not result in a difference in LQ between mice with the control virus (group 2) and those with ChR2 but incorrectly placed cannulae (group 5; simple main effect, *p* = 0.98; Fig. 4).

Finally, photostimulation in mice expressing ChR2 with properly placed fiber optic cannulae (group 1) significantly attenuated the LQ as compared to the pre-test condition of both mice with the control virus (group 2, simple main effect, *p* = 0.002) and those mice with incorrect fiber optic placement (group 5, simple main effect, *p* = 0.03). All mice, regardless of viral injection, that received subcutaneous oil injections without hormones were uniformly non-receptive (LQ = 0; data not shown).

### MOR is internalized in MPN neurons following photostimulation of β-End terminals

**Figure 5.**
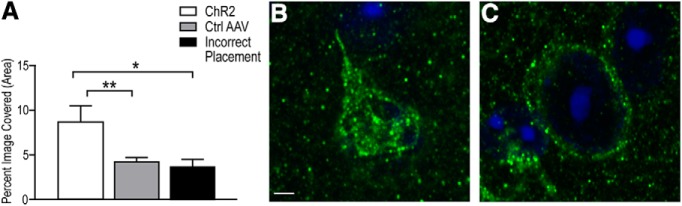
Photostimulation of ChR2 in β-End terminals in the MPN internalizes the MOR. Photostimulation induced the internalization of MOR within cells in the MPN. ***A***, A one-way ANOVA indicated that there was a significant difference in receptor internalization between groups (*F*_(2,11)_ = 5.15, *p* = 0.03, *r* ^2^ = 0.48). Tukey’s multiple comparisons test indicated that mice with ChR2 in β-End terminals in the MPN showed a greater percentage of the area of the image covered by MOR-ir (mean, 8.7 ± 1.8%) as compared to both mice that received the control AAV (mean, 4.3 ± 0.4%; *p = 0.05) and those with ChR2 but incorrect fiber optic placement (mean, 3.7 ± 0.4%, **p = 0.04). Images show DAPI (blue) and MOR (green) in a representative cell from group 1 (***B***) and group 2 (***C***). Scale bar = 2 µm. All values expressed as mean ± SEM.

A one-way ANOVA followed by Tukey’s multiple comparisons test detected a significant difference in receptor internalization (*F*_(2,11)_ = 5.15, *p* = 0.03, *r* ^2^ = 0.48; Fig. 5) measured as the percentage of the area of the image expressing MOR-ir. Pomc-cre mice expressing ChR2 (group 1) showed an increase in receptor internalization (mean, 8.7 ± 1.8%) as compared to both mice with the control virus (group 2; mean, 4.3 ± 0.4%; *p* = 0.05) and mice with incorrectly placed cannulae (group 5; mean, 3.7 ± 0.8%, *p* = 0.04) in response to photostimulation. There was no difference in receptor internalization between group 2 and group 5 (*p* = 0.94).

### cFOS is activated in tdTomato-expressing soma in the ARH

Following behavioral testing, cFOS-ir was evaluated to determine neuronal activation of ARH POMC neurons. In all mice, cFOS-ir occurred only in a subset of POMC neurons in the ARH.

**Figure 6.**
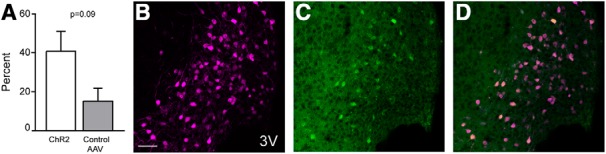
cFOS expression in the ARH is increased following photostimulation. IHC for cFOS was run to confirm activity in POMC neurons in the ARH. ***A***, Following photostimulation, cFOS trended toward greater expression in neurons in mice with ChR2 than those that had the control virus (ChR2 mean, 41.3 ± 9.6%; control AAV mean, 15.7 ± 6.2%; *p* = 0.09), though this effect was not statistically significant, likely due to a lack of statistical power. tdTomato in magenta (***B***), cFOS in green (***C***), and merged image with both tdTomato and cFOS (***D***) in a representative mouse expressing ChR2 in the ARH. Double-labeled neurons appear yellow. Scale bar = 50 µm. 3V, 3rd ventricle. All values expressed as mean ± SEM.

In response to photostimulation, mice with ChR2 trended toward expressing a greater percentage of cFOS in tdTomato-positive neurons (mean, 41.3 ± 9.6%) than did mice with the control virus (mean, 15.7 ± 6.2%), although the effect was not statistically significant (unpaired *t* test, ChR2 vs control AAV, *t*_(5)_ = 2.021, *p* = 0.09; Fig. 6). Furthermore, cFOS-ir occurred almost exclusively in POMC neurons.

## Discussion

In this study, we selectively activated the POMC population involved in sexual receptivity behavior by injecting a ChR2-AAV into the ARH of Pomc-cre mice and subsequently photostimulating the β-End-releasing terminals in the MPN. We found that the photostimulation of these terminals attenuated lordosis behavior in hormone-primed, sexually receptive mice. These results support the hypothesis that activation of MOR is involved in the inhibition of lordosis behavior seen following estradiol administration (Eckersell et al., 1998; Sinchak and Micevych, 2001; Mills et al., 2004). Furthermore, the data suggest that activation of these receptors is capable of over-riding hormonal signals that would otherwise facilitate lordosis.

Only Pomc-cre mice expressing ChR2 showed a significant decrease of the LQ in response to photostimulation compared with mice in all other conditions, including their own pre-test. Interestingly, this inhibition of lordosis behavior was comparable to that seen in mice with a global MOR knock-down (Sinchak et al., 2005), which is a strong indication that an important modulatory aspect of sexual receptivity is mediated through MOR activation in the MPN. The role of MOR in the lordosis circuit has been supported by pharmacological studies indicating that the application of β-End (Wiesner and Moss, 1984; Torii et al., 1996, 1999) or other MOR agonists (Sinchak and Micevych, 2001; Acosta-Martinez and Etgen, 2002a,b) reduces the expression of lordosis. Blocking MOR activation/internalization with antagonists is sufficient to block the estradiol-induced MOR inhibition of sexual receptivity (Wiesner and Moss, 1984), even in the presence of an agonist (Sinchak and Micevych, 2001).

In gonadally intact rodents, estradiol from maturing ovarian follicles activates the ARH – MPN circuit, inhibiting lordosis (for review, see Micevych and Meisel, 2017). To coordinate the expression of lordosis behavior with ovulation, P4 relieves the MOR-mediated inhibition, allowing sexual receptivity to be synchronous with ovulation maximizing reproductive potential. In the present experiment, ovx mice were treated with estradiol and P4 to mimic the sequential ovarian hormonal stimulation that produce a sexually receptive female. Under these conditions, MOR are not activated and are localized primarily on the cell membrane. As expected, only mice in group 1 (Table 1) showed a decrease in the LQ in response to photostimulation (Fig. 4), and this inhibition of behavior was coincident with an increase in MOR internalization (Fig. 5), which follows receptor activation (Sinchak and Micevych, 2001; Micevych et al., 2003; Al-Hasani and Bruchas, 2011), not seen in any other group. Mice that remained sexually receptive had MOR on the cell surface, an indication that these receptors were not activated.

While receptor activation and internalization are two separate events, internalization has been established as an assay for receptor activation (Eckersell et al., 1998 ; Sinchak and Micevych, 2003; Mills et al., 2004). MOR is a G protein-coupled receptor (Manglik et al., 2012), coupled to inhibitory G proteins (Al-Hasani and Bruchas, 2011). MOR agonists (i.e., morphine, endomorphin-1) activate these receptors in the MPN, which results in their internalization (Sinchak and Micevych, 2001; Micevych et al., 2003), and inhibits lordosis behavior (Sinchak and Micevych, 2001), but so does estradiol treatment (Mills et al., 2004). Furthermore, estradiol decreases the firing rate of neurons in the medial preoptic area (Bueno and Pfaff, 1976), a region that includes the MPN and has a high concentration of MOR (Hammer, 1984). Our data here show that the specific activation of these receptors is sufficient to attenuate lordosis behavior. Taken along with the initial inhibitory actions on this circuit, the data suggests that estradiol acts in part on an inhibitory circuit to constrain sexually receptive behavior. Ultimately, MOR is a key component of the circuit controlling lordosis behavior following estradiol treatment (Eckersell et al., 1998; Sinchak and Micevych, 2003; Mills et al., 2004).

Furthermore, in gonadally intact rodents, the internalization of MOR varies across the estrous cycle. Receptor density is highest during diestrus, when the animal is unreceptive, and lowest during proestrus (Hammer, 1984, 1990). The estradiol-induced internalization of MOR is regulated by ERα. In mice with a global knock-out (KO) of ERα (ERαKO), estrogen does not induce the internalization of MOR, though estradiol does induce receptor internalization in ERβKO mice (Micevych et al., 2003). However, these ERαKO mice still exhibit MOR internalization in response to direct MOR agonists (Micevych et al., 2003), indicating that the MOR is still functional Additionally, ERα is found in a subset of POMC neurons (Morrell et al., 1985) and estradiol regulates the expression of the β-End peptide (Priest and Roberts, 2000). Like MOR, β-End fluctuates across the estrous cycle (Barden et al., 1981; Wardlaw et al., 1982).

Following estradiol, P4 relieves the opioid-induced inhibition and MORs are no longer activated, as indicated by their presence on the cell surface, thereby allowing the display of lordosis behavior (Sinchak and Micevych, 2001). This is confirmed by the observation that in all mice except for those that expressed ChR2 and received photostimulation (group 1; Table 1), P4 was sufficient to allow MOR to return to the cell surface, and these mice displayed lordosis. Our finding that photostimulation of ChR2 in the MPN resulted in the internalization of the receptor and inhibition of lordosis, even with P4 in the system, supports previous findings that the activation of MOR is powerful enough to inhibit the hormonal facilitation of lordosis.

Importantly, mice with ChR2 and correct fiber optic placement showed a statistically significant decrease in the LQ compared with those mice with ChR2 but where the cannulae were not positioned in the medial MPN (Fig. 4). The intensity of laser light decreases in an approximately linear fashion as it propagates from the source (Britt et al., 2012). Of the mice with incorrect placement, the average distance of placement was ∼1.5 mm away from the MPN. Using the Predicted Irradiance Values calculations provided by the Diesseroth Lab (https://web.stanford.edu/group/dlab/cgi-bin/graph/chart.php), the irradiance value falls from 7.95 mW/mm^−2^ immediately at the source to 0.06 mW/mm^−2^ at a distance of 1.5 mm. This decrease in photostimulation was not sufficient to stimulate POMC terminals to release β-End to either activate/externalize MORs or impact lordosis behavior. There was a significant difference (*p* = 0.04) in MOR internalization between ChR2-expressing mice with correct optical fiber placement and incorrect placement.

POMC IHC was used to verify that tdTomato expression occurred in POMC neurons. POMC-ir was colocalized with the fluorescent reporter tdTomato (Fig. 2), but a large number of neurons expressed tdTomato alone without POMC-ir, findings similar to Padilla et al., 2012. Within the ARH, tdTomato was expressed in the majority of cell bodies as indicated by the fluorescent reporter tdTomato, but many of these labeled neurons did not express Alexa Fluor 488. However, cFOS-ir occurred only in a subset of these POMC neurons (Fig. 6), rather than the entire population. This likely represents activation of only the reproductively-relevant POMC neurons. While the increase in cFOS-ir likely occurred at least in part in response to mounting by the male (Pfaus et al., 1993), it is possible that at least some of the activation occurred in response to photostimulation in the MPN (Stamatakis and Stuber, 2012), as cFOS-ir in the ARH trended toward being greater in mice with ChR2 than with the control virus.

Both the optical stimulation parameters and MOR internalization provide a level of certainty that the effect on lordosis behavior was due to release of β-End in the MPN. Although we did not formally measure β-End release (i.e., microdialysis; Al-Hasani et al., 2018), stimulation with 20 Hz has been demonstrated to release neuropeptides, in contrast to a lower frequency stimulation (i.e., 2 Hz) that releases amino acid transmitters (Arrigoni and Saper, 2014). In the present study, 20 Hz stimulation activated POMC/β-End terminals in the MPN. We expected that this stimulation would produce a rapid release of either GABA or glutamate, transmitters expressed in POMC neurons (Hentges et al., 2009), followed by longer lasting peptide-mediated response. However, to ascertain that β-End was released, we assessed MOR internalization – an indicator of receptor activation. As expected, following stimulation the pattern of MOR internalization was coincident with inhibition of lordosis and consistent with previous pharmacological studies that indicated that opiate and endogenous opioid activation of MOR in the MPN inhibits lordosis (Pfaus and Pfaff, 1992; Eckersell et al., 1998; Torii et al., 1996, 1999; Sinchak and Micevych, 2001; Mills et al., 2004; Sinchak et al., 2005; Long et al., 2017). At this point, we cannot determine whether the release of amino acid transmitters from POMC terminals also affects the lordosis response.

Overall, several converging lines of evidence suggest that it is β-End release that accounted for the behavioral results: (1) photostimulation was effective only in mice that both expressed ChR2 and had correct fiber placement; (2) cFOS-ir was localized in a subset of POMC-expressing ARH neurons; (3) the decrease in lordosis behavior was coincident with increased MOR internalization in the MPN.

The present study is a refinement of previous methodologies designed to investigate the role of the β-End in reproduction. The use of optogenetics allows for highly specific activation of β-End terminals only in the MPN, rather than pharmacological studies which may inadvertently affect receptors in regions outside of the MPN. The MOR (along with delta and kappa opioid receptors) are found throughout the brain (Mansour et al., 1987, 1988; Erbs et al., 2015). Indeed, the MOR is found in circuits both within and outside of the hypothalamus (Mansour et al., 1987, 1988) that may directly and indirectly affect reproduction. For example, intracerebroventricular infusion of MOR agonists and/or antagonists (Pfaus and Pfaff, 1992) may affect opioid receptors throughout the brain and even the periphery, depending on their half-life (Pardridge, 2011). Moreover, peripheral administration of MOR agonists ( Wiesner and Moss, 1984) likely have effects on non-reproductive functions that may confound experimental interpretation. In this study, we specifically activated only those β-End terminals that expressed ChR2 in the MPN, thereby reducing the potential for unwanted non-specific effects on the MOR, and other opioid receptors.

The full expression of lordosis requires both MOR activation (Sinchak et al., 2005) and a period of behavioral inhibition (for review, see Micevych et al., 2017). Our results indicate that photostimulation of ChR2 in POMC/β-End terminals in the MPN is sufficient to both attenuate lordosis and activate/externalize the MOR in a hormone-primed, sexually receptive female mouse. Thus, taken with previous studies, our data confirm the important inhibitory role that the MOR plays in regulating lordosis behavior.

While humans do not exhibit lordosis as a reproductive reflex, the use of opioids has been linked to the inhibition of reproductive function in humans (Vuong et al., 2010; Grover et al., 2014) and suggests an inhibitory role for these peptides in reproduction between species. Given that much of the hypothalamic circuitry for homeostatic and reproductive functions has been conserved within mammals through evolution (Xie and Dorsky, 2017), the further study of the role of opioids in reproduction provides a window into the hypothalamic control of reproduction.

## Synthesis

Reviewing Editor: Margaret McCarthy, Univ of Maryland School of Medicine Decisions are customarily a result of the Reviewing Editor and the peer reviewers coming together and discussing their recommendations until a consensus is reached. When revisions are invited, a fact-based synthesis statement explaining their decision and outlining what is needed to prepare a revision will be listed below. The following reviewer(s) agreed to reveal their identity: Julie Bakker.

The reviewers agree that the aim of the study was to evaluate if optogenetic activation of B-endorphin terminals in the medial preoptic nucleus inhibits sexual receptivity. The study is based on the model that estradiol activates NPY and POMC neurons in the ARC which then project onto MPN neurons expressing the µ opioid receptor (MOR) leading to an internalization of these receptors and by consequence a tonic inhibitory action on target lordosis control neurons in the VMH. Progesterone reverses the estradiol-induced MOR internalization, thereby reducing the inhibition of VMH neurons, which then facilitates the expression of lordosis. Previous work has been conducted in female rats, and thus an important question is whether a similar model can be applied to female mice, which is in part the point of the curret report.

There are two main points the reviewers strongly agree on.

1) The first is the use of pre-screening fro females with an LQ higher than 70%. This means the alternative outcome, that stimulation increases lordosis, is not being tested. It also means the effects only apply to a subset of females which may have latent variable not apparent here. Hormone primed mice that displayed a LQ of less than 70% were considered to be non-receptive. When looking at the mouse literature, in has been reported that LQ's can vary between 50 and 80% (this is sharp contrast with rats which show much higher LQ's), so this experiment has been conducted on a sub population of extremely receptive female mice. Raising the question of whether an inhibition would have been observed if the more general mouse population has been used. The reviewers and I agree that additional experiments on females with LQ's over a wider range would greatly strengthen the manuscript.

2) The second major point of concern is that only approximately 30% of tdTomato-positive neurons (proof of viral infection) actually expressed POMC in POMC-Cre mice. This raises concerns regaring the specificity of the Cre mouse model. So clearly other additional neuronal populations in the ARC have been infected with the ChR2 virus and some of these might also project to the MPN.

There was also considerable concern about the statistical analyses which stems at least in part from confusion about what the groups are.

1) The group description is confusing, if understood correctly there are 3 groups: Mice transfected with AVV expressing ChR2 and mice with the control virus. Additionally on page 11 the authors mention that “a group of mice (n=4) expressing ChR2 had misplaced fiber optic cannulae, and these animals were used as an additional control group”. However, on table 1 there are 9 groups which description and rationale for each one is not mentioned nor explained in the text. Moreover, how reliable is to have groups with 3 subjects? If there are indeed 3 groups tested in the baseline and in the 3 tests the analysis of the behavioral data and the ICC by multiple t-Tests comparisons is not appropriate. The authors need to run a 3 (group) x 4 (tests) ANOVA with repeated measures. That would significantly reduce the number of figures. For that they need at least 5 subjects per cell. If this is not the case they need to run a Friedman ANOVA for the tests and a Kruskal Wallis for the groups.

There were a number of additional points that require responding to as well.

1.- The inhibitory role of opioids upon lordosis behavior has been postulated some time ago. Although it is important to cite their own work, the authors need to include some of the early studies that postulated this possibility in the introduction and discussion.

Estrogen modulation of mu-opioid receptor-stimulated [35S]-GTP-gamma-S binding in female rat brain visualized by in vitro autoradiography. Acosta-Martinez M, Etgen AM. Neuroendocrinology. 2002 Oct;76(4):235-42.

The role of delta-opioid receptors in estrogen facilitation of lordosis behavior. Acosta-Martinez M, Etgen AM. Behav Brain Res. 2002 Oct 17;136(1):93-102.

Activation of mu-opioid receptors inhibits lordosis behavior in estrogen and progesterone-primed female rats. M Acosta-Martinez, AM Etgen - Hormones and Behavior, 2002 -

2.- According to the authors the POMC neurons project to the medial portion of the MPN, where there is a concentration of cells expressing u-opioid receptors. Are these projections only to the MPN or to the MPOA.? Moreover, by looking at figures 2 and 3 it is clear that the label is present in the medial preoptic region and is not restricted only to the MPN. So, the title should be change to “the medial preoptic region” and not “medial preoptic nucleus”.

3.- The description of the behavioral test is confusing. The authors mentioned that female mice were subject to a pre-test to determine sexual receptivity in a plexiglass testing arena. Then they mentioned that the optogenetic patch cord was attached and testing began. In the same section they describe that photostimulation was applied for the duration of a second behavior test. At the end of the paragraph the authors mentioned that females were exposed to a male once prior to behavior testing and 3 subsequent behavioral tests. The experimental procedures are not very well-described. Mice were first pre-tested, then removed and re-tested with lights on? Were mice actually tested with a male before to induce considerable levels of receptive behavior?

4.- The duration on baseline and the 3 behavioral tests was the same? 10 mounts or 15 min?

What was the rationale or the differences for each one of the 3 behavioral tests? During the baseline and the 3 tests was the same male used? What happened if he ejaculated?

5.- The authors explain the rationale for the POMC IHC, but nothing is mentioned about the C fos IHC. Although it might be obvious it would be good to mention the rationale in the introduction or methods section.

6.- Why are the same results are presented in Figures 4 and 5 and actually in the other Figures as well, there is a redundant presentation of the same results, not adding anything important to the paper

7.-The statement in the first paragraph of the discussion is an overstatement: “We found that the photostimulation of these terminals inhibited the full expression of lordosis behavior in hormone-primed, sexually receptive mice. A LQ of 52% can still be considered as “receptive”, there is no such thing as “full expression of lordosis behavior”. Also it is unclear how this is in line with the hypothesis that activation of the MOR is involved in the inhibition of lordosis behavior seen following estradiol administration since experimental mice were treated with both estradiol and progesterone in the present study.

8. - The quality of the photomicrographs (Figures 2 and 11) could be improved as it is hard to determine double-labeling.

## Author Response

I am pleased to inform you that your manuscript, “Optogenetic activation of β-endorphin terminals in the medial preoptic nucleus regulates sexual receptivity,” has been judged potentially suitable for publication in eNeuro, provided appropriate revisions are made. The decision was a result of the reviewers and me coming together and discussing our recommendations until a consensus was reached. I have put together a fact-based synthesis statement explaining the decision and appended it to this email.

It is important that you revise your manuscript to address the reviewers' concerns, and submit a point-by-point reply to the reviews that indicates your response to each concern. Before a final decision about publication is made, we will have your revision re-reviewed by one or both reviewers.

New: eNeuro is launching a new effort to improve rigor, transparency, and reproducibility, with a strong encouragement to use estimation statistics. Please refer to the editorial

https://www.eneuro.org/content/6/4/ENEURO.0259-19.2019 and commentary

https://www.eneuro.org/content/6/4/ENEURO.0205-19.2019 for the why and how. If the use of estimation statistics applies to your research, you may consider including it for the revision. We will regularly highlight these papers to the community to show how helpful estimation statistics can be, increasing the exposure of your work.

Your revision must include the manuscript with new text indicated in a bold or colored font to aid the re-review of the manuscript. Separately, please provide a clean copy of the manuscript that includes the title page which will be published if accepted. Please closely review your manuscript at this time for any final corrections in style or substance. If your revision is accepted, you will not be allowed to make style or content changes at the proof stage.

At eNeuro we value the contributions of individual authors and want to offer the opportunity to better acknowledge the roles of all authors. As of December 2018, corresponding authors may include specific individuals' roles to data acquisition and analysis at the end of each figure legend. For more information, please see: http://www.eneuro.org/content/preparing-manuscript#fig_legends

Your submission must also include publication-quality figures, each in a separate EPS or TIFF (300 dpi) file, if applicable. Please make sure your figures adhere to the style requirements to avoid delays in manuscript processing. Detailed guidelines for figures are available in our Information for Authors found here: http://www.eneuro.org/content/information-authors

If you would like to include a visual abstract with your revision, you can refer to instructions found here: http://www.eneuro.org/content/preparing-manuscript#visual

When uploading the revised manuscript, there is a link available for the corresponding author to complete the electronic License to Publish form. A link to the form will also be available on the author's home page at http://eNeuro.msubmit.net if not completed at time of submission.

Please return your revision within 3 months of this decision. If you need more time, please contact eNeuro@SfN.org. A checklist for submitting a revision can be found in our Information for Authors here: http://www.eneuro.org/content/submitting-manuscript

When you are ready to submit your revision, you can log in using the link below and click on the manuscript number to create your revision. If you have any questions or concerns, please contact eNeuro@SfN.org.

https://eneuro.msubmit.net/cgi-bin/main.plex

Synthesis of Reviews:

Computational Neuroscience Model Code Accessibility Comments for Author (Required):

N/A

Significance Statement Comments for Author (Required):

appropriate

Comments on the Visual Abstract for Author (Required):

N/A

Synthesis Statement for Author (Required):

The reviewers agree that the aim of the study was to evaluate if optogenetic activation of B-endorphin terminals in the medial preoptic nucleus inhibits sexual receptivity. The study is based on the model that estradiol activates NPY and POMC neurons in the ARC which then project onto MPN neurons expressing the µ opioid receptor (MOR) leading to an internalization of these receptors and by consequence a tonic inhibitory action on target lordosis control neurons in the VMH. Progesterone reverses the estradiol-induced MOR internalization, thereby reducing the inhibition of VMH neurons, which then facilitates the expression of lordosis. Previous work has been conducted in female rats, and thus an important question is whether a similar model can be applied to female mice, which is in part the point of the current report.

There are two main points the reviewers strongly agree on.

1) The first is the use of pre-screening for females with an LQ higher than 70%. This means the alternative outcome, that stimulation increases lordosis, is not being tested. It also means the effects only apply to a subset of females which may have latent variable not apparent here. Hormone primed mice that displayed a LQ of less than 70% were considered to be non-receptive. When looking at the mouse literature, in has been reported that LQ's can vary between 50 and 80% (this is sharp contrast with rats which show much higher LQ's), so this experiment has been conducted on a sub population of extremely receptive female mice. Raising the question of whether an inhibition would have been observed if the more general mouse population has been used.

• The reviewers are correct, the alternative hypothesis is not being tested in this study. Our stated hypothesis is that activation of POMC/β-End terminals in the medial portion of the medial preoptic nucleus inhibits lordosis. Previous data has indicated that MOR activation inhibits lordosis behavior (Torii et al., 1999, Sinchak and Micevych 1991, Mills et al., 2004). These previous experiments using passive immunoneutralization, pharmacological antagonism, and MOR internalization assays all pointed to an inhibition of behavior. Thus, the experiment was designed to test the selective activation of arcuate nucleus PMOC/β-End terminals in receptive females. We acknowledge that an experiment to examine POMC/β-End activation in non- to moderately-receptive animals would be interesting, but this was not the design of the present experiment

• For this study, and most studies in our hands, we chose to use and LQ of 70 as the cutoff point. This is for several reasons, the first of which being that we find an LQ of 50 no better than chance, therefore we can't be sure of the results we're seeing. Additionally, we wanted to ensure that mice were receptive so that we could see an effect (if there was to be one). Furthermore, this study builds upon previous studies within our lab, therefore the intention was also to remain consistent with previous experimental design. We agree, however, that lordosis in mice is much less consistent than lordosis in rats, and that many other groups include a wider range of LQ scores within their definition of receptive.

• Data from all mice that underwent lordosis testing was recorded. To address the question of whether this effect is seen in the greater population we reviewed our data. Lowering the threshold of what we consider receptive to include mice with an LQ of 50, the data indicate that photostimulation decreases the LQ of these mice as well. If we include these mice in Group 1 (Cre+, ChR2, hormones), photostimulation decreased the LQ from 79.86 {plus minus} 3.3 in the pre-test to 52.57 {plus minus} 9.0 during stimulation (t(6)=3.659, p=0.01). This is compared to a pre-test LQ of 81.4 {plus minus} 3.0 versus a photostimulated LQ of 52.9 {plus minus} 9.5 (t(6)=3.564, p=0.01) in mice included in the manuscript. Therefore, given that there was no statistical difference when these LQ scores were included, we would like to keep the threshold for receptivity as it is in the original manuscript.

2) The second major point of concern is that only approximately 30% of tdTomato-positive neurons (proof of viral infection) actually expressed POMC in POMC-Cre mice. This raises concerns regarding the specificity of the Cre mouse model. So clearly other additional neuronal populations in the ARC have been infected with the ChR2 virus and some of these might also project to the MPN.

This is, unfortunately, a noted limitation of the mouse model used, which we attempted to address experimentally as well as through published literature (see citation, as well as Morrison & Münzberg, 2012). Populations that do not express POMC in the adult may still express Cre through activity of the Pomc promoter during development. Unfortunately, when the study began this was the best model that was available. If other (non-POMC) populations do in fact express Cre, an interesting next step in this study could be to address this issue more specifically and to follow up on which, if any, other neurotransmitters or peptides might be release, and if they interact with MOR/lordosis. We feel confident that the reported effect was due to release of β-End because of the coincidence of MOR internalization/activation - an indication that an endogenous opioid had activated the MOR. A final additional explanation, though perhaps less likely, is that it is also possible that the POMC antibody underestimated the number of POMC-positive neurons.

There was also considerable concern about the statistical analyses which stems at least in part from confusion about what the groups are.

1) The group description is confusing, if understood correctly there are 3 groups: Mice transfected with AVV expressing ChR2 and mice with the control virus. Additionally on page 11 the authors mention that “a group of mice (n=4) expressing ChR2 had misplaced fiber optic cannulae, and these animals were used as an additional control group”. However, on table 1 there are 9 groups which description and rationale for each one is not mentioned nor explained in the text. Moreover, how reliable is to have groups with 3 subjects? If there are indeed 3 groups tested in the baseline and in the 3 tests the analysis of the behavioral data and the ICC by multiple t-Tests comparisons is not appropriate. The authors need to run a 3 (group) x 4 (tests) ANOVA with repeated measures. That would significantly reduce the number of figures. For that they need at least 5 subjects per cell. If this is not the case they need to run a Friedman ANOVA for the tests and a Kruskal Wallis for the groups.

• The groups and descriptions have now been clarified in the text and the corresponding tables. There were several groups included in this study, most of which were controls. The groups of importance are the following:

o Group 1: Cre(+), received ChR2, received hormones (main experimental group)

o Group 2: Cre(+), received control virus, received hormones (direct control for above group)

o Group 5, originally Group 9: Cre(+), received ChR2, received hormones BUT also received misplaced fiber optic cannula (a control for anatomical specificity of MOR activation).

• The following groups served as additional controls, which are perhaps unnecessary as the effects of hormones are a) understood, and b) not the focus of this study.

o Group 3: Cre(+), received ChR2, oil control

o Group 4: Cre(+), received control virus, oil control

• Finally, the following groups as additional controls, to verify that the virus was specific to cre(+) neurons.

o Group 5 (removed): Cre(-), received ChR2, received hormones

o Group 6 (removed): Cre(-), received control virus, received hormones

o Group 7 (removed): Cre(-), received ChR2, oil control

o Group 8 (removed): Cre(-), received control virus, oil control.

• The final two groups (7 & 8) were the groups containing only 3 subjects. As these groups, and moreover the entire last 4 groups, were additional, potentially unnecessary, controls whose inclusion provided no additional or relevant information, we have decided to remove them from the manuscript. This also simplifies the misunderstanding regarding choice of statistics. However, if the reviewers believe these final 4 groups (or some subset of them) should be included, we can replace them.

• As we understand the comment regarding statistics, the suggestion is to treat each behavior test as a separate condition, and to include the IHC as a test (4 tests), by group (3 as understood by the reviewers). As we designed it, the 3 sets of behavioral tests (pre/post) were run to get an average LQ/mouse (because in our hands, and other labs it seems, mice display less consistent lordosis responses than do rats), not as a means to test changes in behavioral responses over time, to result in a single pre-test LQ and a single photostimulated LQ per mouse. As written, it was unclear in the text that we averaged the results, and that has since been clarified. This may also clarify the choice of statistics used. Additionally, the IHC was run to correlate MOR internalization with the photostimulation condition, not to be statistically analyzed immediately with the behavioral tests.

• All statistics have been updated to reflect the removal of unnecessary groups, and to reflect combining some of the figures, as noted further along in the comments.

There were a number of additional points that require responding to as well.

1.- The inhibitory role of opioids upon lordosis behavior has been postulated some time ago. Although it is important to cite their own work, the authors need to include some of the early studies that postulated this possibility in the introduction and discussion.

Estrogen modulation of mu-opioid receptor-stimulated [35S]-GTP-gamma-S binding in female rat brain visualized by in vitro autoradiography. Acosta-Martinez M, Etgen AM. Neuroendocrinology. 2002 Oct;76(4):235-42.

The role of delta-opioid receptors in estrogen facilitation of lordosis behavior. Acosta-Martinez M, Etgen AM. Behav Brain Res. 2002 Oct 17;136(1):93-102.

Activation of mu-opioid receptors inhibits lordosis behavior in estrogen and progesterone-primed female rats. M Acosta-Martinez, AM Etgen - Hormones and Behavior, 2002 -

• The suggested citations are appreciated, and have been added as appropriate. The second citation that was suggested has not been included. The content of the paper, while interesting and informative, does not fit with the scope of the current research, which was not intended to be a comprehensive overview of all opioid receptors involved in reproduction, but rather to focus more specifically on the MOR in the MPN.

2.- According to the authors the POMC neurons project to the medial portion of the MPN, where there is a concentration of cells expressing u-opioid receptors. Are these projections only to the MPN or to the MPOA.? Moreover, by looking at figures 2 and 3 it is clear that the label is present in the medial preoptic region and is not restricted only to the MPN. So, the title should be change to “the medial preoptic region” and not “medial preoptic nucleus”.

• The reviewers are correct, fibers from POMC/tdTomato-expressing neurons in the ARH do innervate the general Medial Preoptic Area (MPOA). However, it is also true, as noted, that there is a concentration of MOR-expressing cells in the medial MPN, which have been shown to be involved in the expression of lordosis behavior. This is the particular sub-region tested in this experiment, not the greater MPOA. Moreover, when fiber optic cannulae were misplaced they still were within the MPOA. Photostimulation within the MPOA, but outside of the MPN, had no effect on lordosis behavior nor on the internalization of MOR. Therefore, we believe the title should remain as submitted, as this was the specific region tested- MOR in the MPN.

3.- The description of the behavioral test is confusing. The authors mentioned that female mice were subject to a pre-test to determine sexual receptivity in a plexiglass testing arena. Then they mentioned that the optogenetic patch cord was attached and testing began. In the same section they describe that photostimulation was applied for the duration of a second behavior test. At the end of the paragraph the authors mentioned that females were exposed to a male once prior to behavior testing and 3 subsequent behavioral tests. The experimental procedures are not very well-described. Mice were first pre-tested, then removed and re-tested with lights on? Were mice actually tested with a male before to induce considerable levels of receptive behavior?

• The description of behavioral testing has been clarified in the text (Methods section). In our hands, the first encounter a female mouse has with a male typically results in little to no lordosis behavior (i.e., LQ of 0-10 out of 10 mounts). Therefore, an initial introduction to a male as a training session, similar to required training in other behavioral tests, is necessary before being able to consistently collect data. Following this initial training session, the female is then tested the 3 subsequent times mentioned in the text.

4.- The duration on baseline and the 3 behavioral tests was the same? 10 mounts or 15 min? What was the rationale or the differences for each one of the 3 behavioral tests? During the baseline and the 3 tests was the same male used? What happened if he ejaculated?

• We have updated this in the text. The key point in our behavioral protocol is 10 mounts by the male, with the allowance to take up to 15 minutes for this to happen. However, very rarely (if ever) did the male take 15 minutes to mount- almost all trials were completed within 5-7 minutes. The time component has been removed from the text, as it really wasn't relevant. If the male ejaculates, the female is moved to another cage containing a different male to continue the trial, until she receives 10 mounts. However, this almost never was the case, as we were typically able to prevent the male from intromitting during behavioral trials.

5.- The authors explain the rationale for the POMC IHC, but nothing is mentioned about the C fos IHC. Although it might be obvious it would be good to mention the rationale in the introduction or methods section.

• This has now been clarified in the text (Methods).

6.- Why are the same results are presented in Figures 4 and 5 and actually in the other Figures as well, there is a redundant presentation of the same results, not adding anything important to the paper.

• Figures 4 and 5 present different, but related, data. However, the confusion is understandable. Originally, Figure 4 presented in (A) the results of pre-test versus photostimulation in ChR2-expressing mice, while (B) presented the results of pre-test versus photostimulation in mice with the control virus. Figure 5 proceeded to then break these groups apart and look at, in (A), the pre-test LQ between ChR2-expressing and control mice, and then (B) LQ of each of these groups in response to photostimulation. These two figures have been combined to present a more cohesive picture of the data. Figure 6 has been renumbered to Figure 5.

• Figure 7 has been removed, as per discussion of the groups in the first point of review. The reasoning for this is because, as the reviewers noted, some data was redundant and provided nothing new in the context of the study. Including Cre-negative animals provided no new information, and seems only to contribute to confusion regarding the data. However, as noted, if the reviewers think this information belongs in the paper, it can be replaced.

• Additionally, Figure 8 (now Figure 6) has been updated to show only 3 groups, not 5, following the removal of Cre-negative mice. This update has been reflected in the text.

7.-The statement in the first paragraph of the discussion is an overstatement: “We found that the photostimulation of these terminals inhibited the full expression of lordosis behavior in hormone-primed, sexually receptive mice. A LQ of 52% can still be considered as “receptive”, there is no such thing as “full expression of lordosis behavior”. Also it is unclear how this is in line with the hypothesis that activation of the MOR is involved in the inhibition of lordosis behavior seen following estradiol administration since experimental mice were treated with both estradiol and progesterone in the present study.

• As noted, in our laboratory 50% is not considered fully receptive and this study aimed to stay in line with these parameters. The phrase “full expression of lordosis behavior” is established within the field as a way to indicate that maximal receptivity is being reached (Etgen & Acosta-Martinez, Endocrinology, 2003; Daniels et al., J Neurosci, 1999; Ganten & Pfaff, 2012; Micevych et al., Trends in Neuroscience, 2017; Wong et al., PLoS One, 2015; Thornton et al., Physiology & Behavior, 1986), therefore we feel it is appropriate to use in this context, as photostimulation inhibited this maximal receptivity.

• Experimental mice were treated with estrogen and progesterone to ensure maximal receptivity. Studies from our lab and others have established that for ~24 hours following estradiol administration, lordosis is inhibited and, importantly, that MOR is activated, and that these events are linked. Were we to only administer estradiol in this study, we would be unable to demonstrate MOR activation and lordosis inhibition in response to photostimulation (as both would already be occurring). Therefore, progesterone was administered to produce sexually receptive mice, which then received photostimulation to induce activation/internalization of MOR, in a way similar to that induced by estradiol.

8. - The quality of the photomicrographs (Figures 2 and 11) could be improved as it is hard to determine double-labeling.

• The images in Figures 2 & (now) 9 have been updated to present double labeling more clearly. Currently the dpi is >300. If the images still appear unclear please let us know. Additionally, to improve visualization the blue DAPI channel has been removed from Figure 2.
